# Potentially inappropriate medications use among the older patients diagnosed with psychiatric diseases in Saudi Arabia: a cross-sectional study

**DOI:** 10.3389/fmed.2025.1534828

**Published:** 2025-03-03

**Authors:** Mohammed M. Alsultan, Solaiman M. Alhawas, Leena H. Alhajri, Khalid A. Alamer, Abdullah K. Alahmari, Amani M. AlQarni, Feras A. Al-Awad

**Affiliations:** ^1^Department of Pharmacy Practice, College of Clinical Pharmacy, Imam Abdulrahman Bin Faisal University, Dammam, Saudi Arabia; ^2^Saudi Food and Drug Authority, Riyadh, Saudi Arabia; ^3^Department of Clinical Pharmacy, College of Pharmacy, Prince Sattam Bin Abdulaziz University, Al-Kharj, Saudi Arabia; ^4^Family and Community Medicine Department, King Fahd Hospital of the University, Imam Abdulrahman Bin Faisal University, Dammam, Saudi Arabia; ^5^Department of Psychiatry, College of Medicine, Imam Abdulrahman Bin Faisal University, Dammam, Saudi Arabia

**Keywords:** potentially inappropriate medication, Beers criteria, older, aging, psychiatric diseases, mental health disease

## Abstract

**Objective:**

To examine the prevalence of potentially inappropriate medications (PIMs) prescribed among older patients diagnosed with psychiatric diseases and to identify associated factors.

**Methods:**

This cross-sectional study was conducted among older patients who visited outpatient clinics in Saudi Arabia between June 1st, 2019, and May 31st, 2023. PIMs use was estimated using the updated 2019 American Geriatric Society (AGS) Beers criteria. Data were analyzed using chi-square or Fisher's exact test for categorical variables and *t*-test for continuous variables to compare patients with and without PIMs. In addition, the Pearson correlation test was performed between the total number of prescriptions and the number of PIMs. Multivariable logistic regression analysis was used to explore PIMs. Statistical significance was set at *p* < 0.05.

**Results:**

Our study included 306 patients with psychiatric diseases, with 156 (50.98%) in the PIMs group and 150 (49.02%) in the non-PIMs group. There was a considerable positive correlation between the total number of prescriptions and the number of PIMs (*r* = 0.76; *p* < 0.0001). The adjusted logistic regression analysis revealed a significantly higher risk of PIMs use in individuals with psychiatric diseases and comorbid neurological diseases compared to those without [adjusted odds ratio (AOR) = 2.48, 95% CI [1.15–5.32]]. In contrast, the risk of PIMs use was not significantly greater for older individuals with psychiatric diseases and comorbid hypertension {AOR = 1.67, 95% CI [(0.79–3.54)]}, diabetes mellitus {AOR = 1.25, 95% CI [(0.66–2.34)]}, or pulmonary disease {AOR = 2.34, 95% CI [(0.69–7.96)]}.

**Conclusion:**

This study highlighted the elevated number of PIMs in older adults with psychiatric diseases in the outpatient setting, particularly those with comorbid neurological diseases. Therefore, clinical pharmacists may play a crucial role in improving the outcomes of patients diagnosed with psychiatric illnesses. Finally, future studies should examine additional strategies to reduce the use of PIMs in this population.

## Introduction

Rational drug therapy is crucial for older adults to effectively manage their health (1). Worldwide, the percentage of aging individuals is projected to increase to 16% by 2050, with a 6% increase compared to 2022 (2). By 2030, older individuals will comprise ~4.63 million of the anticipated total population of the Kingdom of Saudi Arabia, which is projected to be 39.5 million (3).

Unfortunately, up to 20–30% of hospital admissions for older individuals are because of drug-related problems, which frequently result in greater risks of morbidity, mortality, illness, frailty, and higher healthcare costs (4–6). However, chronic illnesses and alterations in the pharmacokinetics and pharmacodynamics, such as delayed renal elimination of drugs and increased sensitivity to anticholinergic and sedating effects are frequently linked to aging (7–9). Additionally, as the prevalence of multimorbidity among older individuals increases, polypharmacy and the use of potentially inappropriate medications (PIMs) are becoming more prevalent in geriatric populations (10). PIMs are sometimes indispensable, necessitating their use despite associated risks. However, PIMs can be replaced with better-tolerated alternatives whose predicted clinical benefits exceed their risk of adverse drug events when administered to older patients (11).

PIMs are frequently occurring medication-related problems that affect older patients. Common scales and criteria are used to evaluate the usage of PIMs, including the Beers criteria, which are a set of precise standards for identifying PIMs that were developed in 1991 and have since been updated (12, 13). Although the factors associated with inappropriate medication use differ, various studies have linked the risk of PIMs to factors such as age, sex, use of multiple medications, and the number of comorbid conditions (14, 15). One systematic review evaluated factors associated with PIMs use in community-dwelling older adults in the United States. The study reported that taking more drugs, female sex, and higher outpatient and emergency department utilization were the most observed statistically significant factors associated with PIMs use (16). Moreover, older adults with specific chronic diseases such as diabetes, hypertension, depression, osteoporosis, and dementia are more likely to use PIMs than those who do not have these diseases (17, 18).

Multiple studies have been conducted to determine the prevalence of and factors associated with PIMs using Beers Criteria. According to the 2012 Beers Criteria, the prevalence of PIMs in European countries ranged from ~22% to 44% (19–21). Additionally, studies conducted in India, the United States, and Brazil indicated that the prevalence of PIMs among older patients ranged from 29.3% to 59.2% (22–24). In Middle Eastern countries, using the 2015 Beers criteria, 62.5% of patients had at least one prescribed PIMs in Jordan, while the application of the 2019 Beers criteria in the Gulf countries indicated a prevalence of ~34.7% to 62.6% (25–28).

In Saudi Arabia, the prevalence of PIMs was reported to be 19% using the 2012 Beers criteria, whereas it increased to around 60% when utilizing the 2015 Beers criteria (29–31). More recent studies using the 2019 Beers criteria indicated that the prevalence of PIMs among individuals with various chronic diseases ranged from 48.6% to 66.6% (32–36). Although these studies have highlighted a significant risk of PIMs in Saudi Arabia for diseases such as diabetes, dyslipidemia, and anxiety, none specifically examined the issue in the context of comprehensive psychiatric diseases. Therefore, this study aimed to investigate the prevalence of PIMs prescribed to older patients in Saudi Arabia who are diagnosed with psychiatric diseases and to identify the associated factors.

## Methods

### Ethical approval

This study was approved by the Institutional Review Board of the Imam Abdulrahman Bin Faisal University, Dammam, Saudi Arabia (IRB-2024-01-200). This study was performed in accordance with the standards of the 1964 Declaration of Helsinki and its later amendments.

### Study design

This was a retrospective cross-sectional study conducted between 1st June 2019, and 31st May 2023.

### Data settings, source, and population

Patient data were extracted from the Electronic Medical Records (EMRs) of older patients who visited the outpatient clinic at King Fahad Teaching Hospital, Khobar, Saudi Arabia. The database contains information on patient demographic variables, such as age, and sex. Data regarding medical diagnoses and prescribed drugs, including the date of each diagnosis, and drugs were extracted. The inclusion criteria were individuals aged 65 years and older, who were diagnosed with at least one psychiatric disease, such as mood disorders, anxiety, schizophrenia, or other psychiatric diseases, based on the International Classification of Diseases, Tenth Revision (ICD-10) codes (Appendix-1).

### Study outcomes

The outcome of our study was to estimate PIMs using the updated 2019 AGS Beers criteria to classify PIMs use. We categorized medications that older adults should avoid or use with caution according to the 2019 Beers criteria. Additionally, we categorized the prevalence of PIMs use into *three* categories: *one* PIM, *two* PIMs, and *three* or more PIMs. Furthermore, PIMs users were classified into *two* categories: PIMs users, who were prescribed at least *one* or more PIMs, and non-PIMs users, who had no prescription for PIMs (13). The covariates included in our study were demographic variables, including age, sex, and comorbidities including neurological diseases, hypertension (HTN), diabetes mellitus (DM), kidney disease, liver disease, dyslipidemia, cancer, and pulmonary disease (Appendix-1). In addition, we evaluated the relationship between the total number of prescriptions and the number of PIMs.

### Data analysis

Descriptive data were utilized to examine the baseline characteristics of patients with PIMs and those without. Categorical variables were presented as frequency and percentage and were assessed using the chi-square or Fisher's exact test, as appropriate. Continuous variables were presented as means and standard deviations (SD) and assessed using student *t*-tests. Pearson correlation (*r*) test was performed to assess the strength and direction between the total number of prescriptions and the number of PIMs. Furthermore, multivariable logistic regression was used to explore PIMs, and the goodness of fit was checked using the Hosmer-Lemeshow test. The adjusted odds ratio (AOR) along with its confidence interval at 95% (95% CI) were presented. The statistical significance was set at *p* < 0.05. The SAS software (version 9.4) was used to analyze the data.

## Results

**Figure 1:**
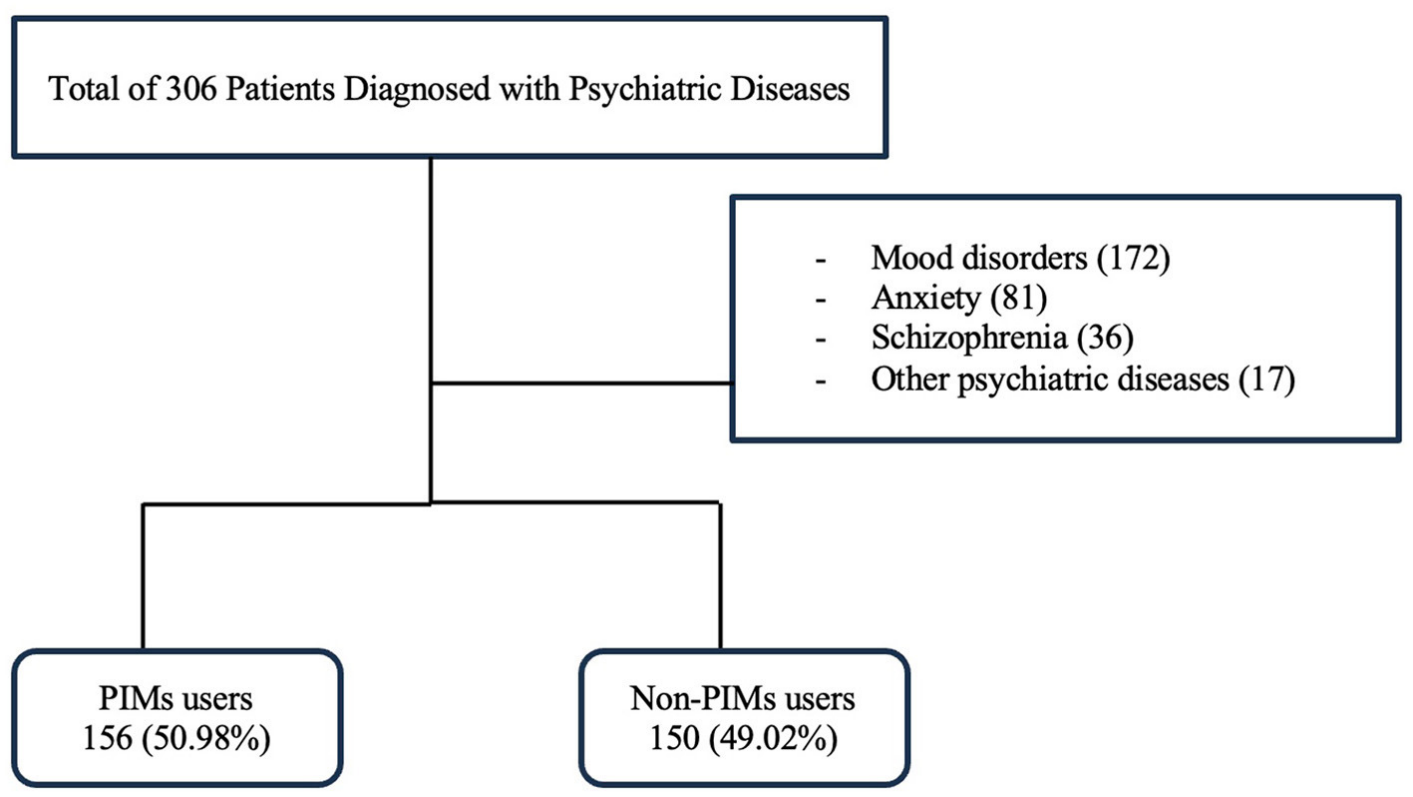
Study flowchart of psychiatric disease patients with and without PIMs.

The total number of individuals in our study was 306 older adults diagnosed with psychiatric disease, with 156 (50.98%) in the PIMs group and 150 (49.02%) in the non-PIMs group, as shown in Figure 1.

The average age of PIMs users was 73.09 ± 6.55 years, whereas non-PIMs users had an average age of 73.87 ± 7.07 years. Among the participants, there were 197 females (64.38%) and 109 males (35.62%), showing no significant difference between the two groups. In addition, PIMs users had a higher rate of various health comorbidities such as neurological diseases (8.17% vs. 4.25%), hypertension (9.80% vs. 6.54%), diabetes mellitus (11.76% vs. 7.84%), liver diseases (2.29% vs. 1.31%), dyslipidemia (4.90% vs. 4.25%), and pulmonary disease (3.92% vs. 1.31%) compared to non-PIMs users, as detailed in Table 1.

**Table 1:** Baseline characteristics of older patients with psychiatric disease using PIMs and non-PIMs.

**Variable**	**PIMs [*N* = 156 (50.98%)]**	**No PIMs [*N* = 150 (49.02%)]**	*P*-value^*^
	***n*** **(%)**	***n*** **(%)**	
**Age, in years—Mean (SD)**	73.09 (6.55)	73.87 (7.07)	0.34
**Sex**			
Female	99 (32.35)	98 (32.03)	0.81
Male	57 (18.63)	52 (16.99)	
**Comorbidities**			
**Neurological diseases**			
Yes	25 (8.17)	13 (4.25)	0.06
No	131 (42.81)	137 (44.77)	
**Hypertension**			
Yes	30 (9.80)	20 (6.54)	0.17
No	126 (41.18)	130 (42.48)	
**Diabetes mellitus**			
Yes	36 (11.76)	24 (7.84)	0.15
No	120 (39.22)	126 (41.18)	
**Kidney diseases**			
Yes	2 (0.65)	6 (1.96)	0.17
No	154 (50.33)	144 (47.06)	
**Liver diseases**			
Yes	7 (2.29)	4 (1.31)	0.54
No	149 (48.69)	146 (47.71)	
**Dyslipidemia**			
Yes	15 (4.90)	13 (4.25)	0.84
No	141 (46.08)	137 (44.77)	
**Cancer disease**			
Yes	3 (0.98)	8 (2.61)	0.13
No	153 (50.00)	142 (46.41)	
**Pulmonary disease**			
Yes	12 (3.92)	4 (1.31)	0.07
No	144 (47.06)	146 (47.71)	

* *p*-value < 0.05.

**Figure 2:**
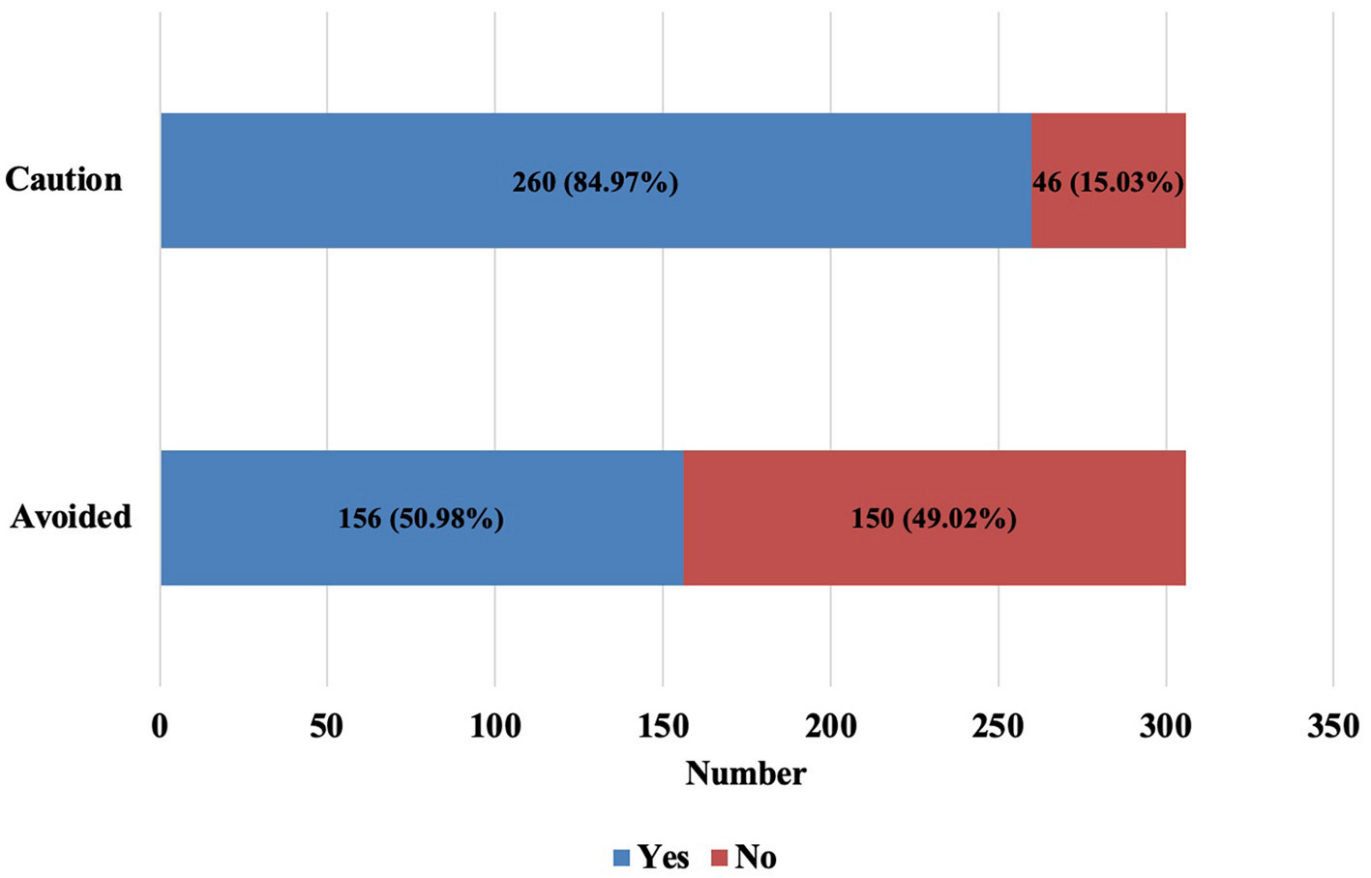
Prevalence of PIMs that should be avoided and used with caution among older with psychiatric diseases.

**Figure 3:**
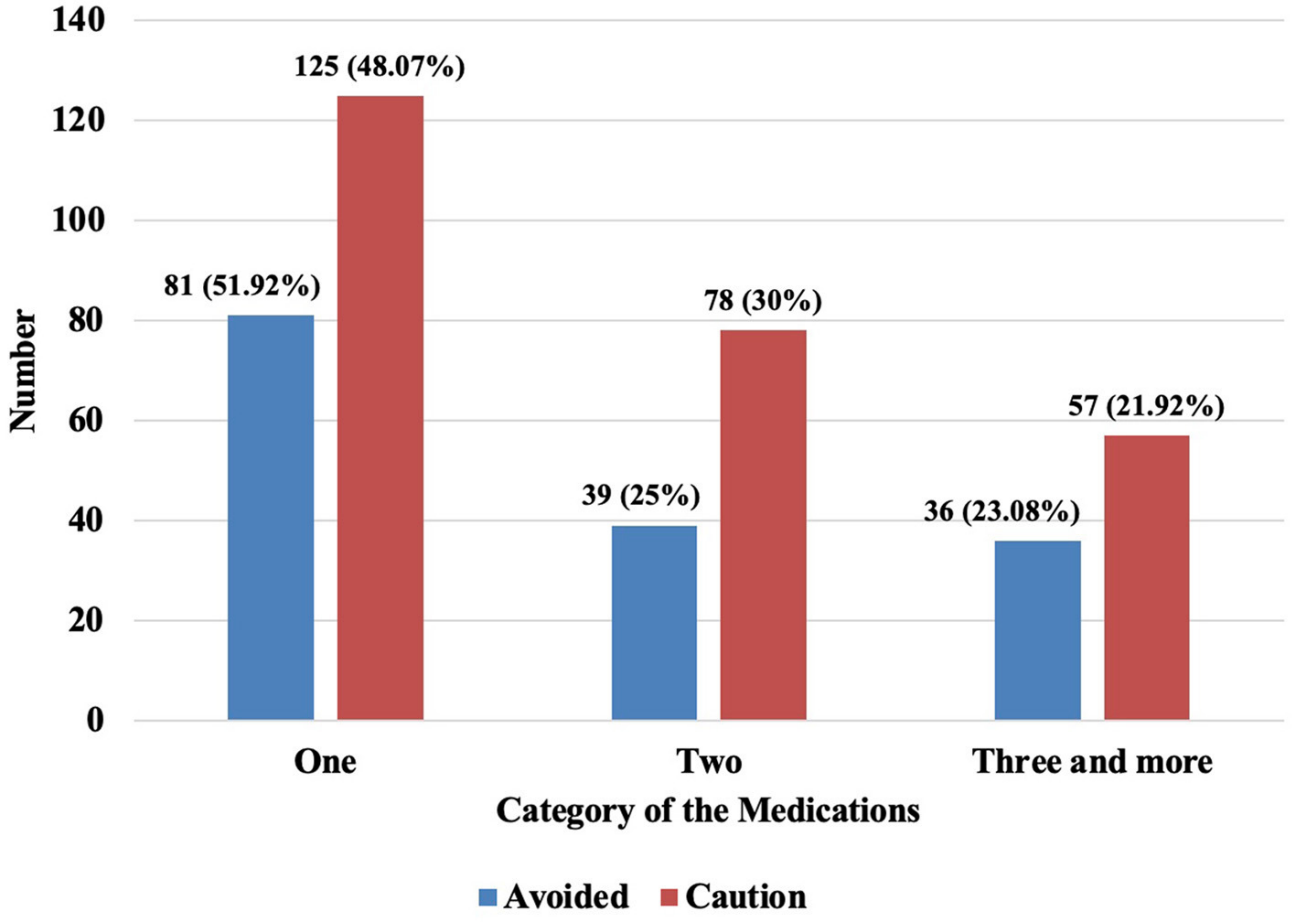
Numbers of PIMs that should be avoided and used with caution among older with psychiatric diseases.

The prevalence of individuals with PIMs that should be avoided in older patients diagnosed with psychiatric disease was (50.98%) whereas the prevalence of individuals with PIMs requiring caution was (84.97%), as shown in Figure 2. Furthermore, the prevalence of medications that should be avoided and used with caution among psychiatric patients was one PIM (51.92% vs. 48.07%), two PIMs (25% vs. 30%), and three or more PIMs (23.08% vs. 21.92%), as displayed in Figure 3. There is a strong positive correlation between the total number of prescriptions and the number of PIMs (*r* = 0.76; *p* < 0.0001).

The most frequently prescribed PIMs to avoid were antipsychotics and antidepressants, while the least prescribed were antimanic, skeletal muscle relaxants, and antihypertensives. On the other hand, we observed that antidepressants, were prescribed more frequently followed by antipsychotic drugs than other classes of medications that should be used with caution (see Table 2). In addition, 45.1% of patients were found to have PIMs because of combination of ≥3 Central Nervous System (CNS)-active drugs.

**Table 2:** Potentially inappropriate medications to be avoided and used with caution among older patients with psychiatric diseases.

**Medication groups**	***N* (%)**
**Should be avoided**	
Antipsychotics	123 (11.51)
Antidepressants	123 (11.51)
Antiepileptic	42 (3.93)
Antiparkinson	23 (2.15)
Gastrointestinal agents	14 (1.31)
Non-benzodiazepine hypnotics	11 (1.03)
Endocrine agents	2 (0.19)
Antimanic	1 (0.09)
Skeletal muscle relaxants	1 (0.09)
Antihypertensive	1 (0.09)
**Use with caution**	
Antidepressants	297 (27.78)
Antipsychotics	184 (17.21)
Antiepileptic	7 (0.65)
Non-opioid analgesics	2 (0.19)

The adjusted logistic regression analysis revealed a significantly higher risk of PIMs use in individuals with psychiatric diseases and comorbid neurological disease compared to those without comorbid neurological diseases (AOR = 2.48, 95% CI [1.15–5.32]). However, increased risk of PIMs was not observed for females (AOR = 0.93, 95% CI [0.57–1.52]), patients with hypertension {AOR = 1.67, 95% CI [(0.79–3.54)]}, diabetes mellitus {AOR = 1.25, 95% CI [(0.66–2.34)]}, kidney diseases {AOR = 0.25, 95% CI [(0.05–1.37)]}, liver diseases {AOR = 1.23, 95% CI [(0.31–4.94)]}, dyslipidemia {AOR = 0.79, 95% CI [(0.32–1.96)]}, cancer {AOR = 0.38, 95% CI [(0.10–1.49)]}, and pulmonary diseases {AOR = 2.34, 95% CI [(0.69 −7.96)]} in comparison to those without these diseases (see Table 3).

**Table 3:** Adjusted logistic regression of potentially inappropriate medications among older patients with psychiatric diseases.

**Predicators**	**AOR**	**95% CI**	
Age	0.97	0.93	1.01
Female	0.93	0.57	1.52
Neurological diseases	2.48	1.15	5.32
Hypertension	1.67	0.79	3.54
Diabetes mellitus	1.25	0.66	2.34
Kidney diseases	0.25	0.05	1.37
Liver diseases	1.23	0.31	4.94
Dyslipidemia	0.79	0.32	1.96
Cancer diseases	0.38	0.10	1.49
Pulmonary diseases	2.34	0.69	7.96

AOR, Adjusted Odds Ratio; CI, Confidence Interval; Hosmer-Lemeshow goodness of fit *p*-value >0.05.

## Discussion

In this study, we assessed the prevalence of PIMs prescribed to older patients in Saudi Arabia diagnosed with psychiatric diseases in the outpatient setting. Our findings indicated that the prevalence of PIMs, which include medications that should be avoided or used with caution was notably high. Psychotropic drugs (i.e., antidepressants and antipsychotics) were the most prescribed PIMs. Most patients with PIMs were found to have ≥3 CNS-active drugs. In addition, the risk of PIMs use was higher among patients with psychiatric disease and comorbid neurological disease.

The prevalence of PIMs in our study was 50.98% for those drugs that should be avoided while 84.97% for drugs that should be used with caution. This high prevalence is in line with other studies of older individuals diagnosed with psychiatric diseases; 63% for medications that should be avoided and 81% for medications that should be used with caution (35, 37). Similar to another study among Saudi Arabian individuals with anxiety disorders, the use of one PIM or less was higher than the use of two or more medicines (35). However, our findings are inconsistent with another study that reported a high prevalence of PIMs (>90%) for drugs that should be avoided or those used with caution. This discrepancy might be related to the study setting (inpatient) and/or study population who had schizophrenia and bipolar disorders (38).

In complex situations, the use of PIMs cannot be avoided in older patients with psychiatric diseases. In other cases, PIMs use can be minimized or replaced with better-tolerated alternatives (11). Previous studies reported at least one potentially inappropriate duplicate therapy or PIMs among 43.1%−46.7% of older patients with psychiatric diseases (39, 40). In addition, drug interactions in older patients with psychiatric diseases were found to be frequently encountered (41). Several studies reported that commonly prescribed antidepressants, antipsychotics, and/or CNS-active drugs were classified as PIMs (42–44). In addition, the updated 2019 AGS Beers criteria strongly recommends avoiding a combination of ≥3 CNS-active drugs (13). This may explain the high number of PIMs identified in our study.

This study highlights the need for urgent interventions to improve medication use in older patients with psychiatric diseases who have comorbid neurological diseases requiring multiple CNS-active drugs. The availability of clinical pharmacists in the primary care setting has been reported to reduce medication prescribing including PIMs in older patients with psychiatric diseases (45). In addition, the interdisciplinary medication reviews led by clinical pharmacists may have a potential role in minimizing the number of PIMs that are not indicated, contraindicated, or have significant drug-drug interactions (46, 47). These interventions are crucial for optimizing patient care for the subgroup mentioned earlier. Additionally, literature reported that preventable adverse drug events constitute a large burden on healthcare expenditure and can be minimized with pharmacist-led medication reviews (48–50). Moreover, counseling older patients on medication management during illness may improve appropriate drug use (51). Future studies should examine the effectiveness of other strategies including the implementation of alerts within computerized provider order entry (CPOE), hospital disease management guidelines, and educational activities in this population (52).

To the best of our knowledge, this is the first study in Saudi Arabia to use the 2019 Beers Criteria and comprehensively include psychiatric diseases in older individuals. Second, the study had a long study duration and explored PIMs use predictors. Finally, this study investigated the number of PIMs used per patient, which should be avoided and used cautiously in older patients with psychiatric diseases. Nonetheless, the study does have some limitations. First, the study was limited to a single center in Saudi Arabia and therefore, the study findings cannot be extrapolated to other centers. Second, the study was limited by its retrospective nature. Since ICD-10 classification has limitations with regard to the exact definition of depression and differentiating between bipolar subtypes, we only included codes with confirmed depression and bipolar diagnoses (53, 54). Third, the EMRs did not record patients' usage of over-the-counter medications, which may have had an impact on the prevalence rate of PIMs use. Finally, the missing data in EMRs may have led to bias in our study.

## Conclusion

This study highlighted the elevated number of PIMs in older adults with psychiatric diseases in the outpatient setting especially those with comorbid neurological diseases. Therefore, clinical pharmacists may play a significant role in improving the outcomes of patients diagnosed with psychiatric diseases. Finally, future studies should examine other strategies to reduce PIMs in this population.

## Data Availability

The datasets presented in this article are not readily available because the data that supports the findings of this study are available from the corresponding author upon reasonable request. Requests to access the datasets should be directed to Mohammed M. Alsultan, mmaalsultan@iau.edu.sa.

## Appendix-1

**Table A1:** ICD-10 codes of the diagnosis and comorbidities.

**Diagnosis**	**ICD-10**
Hypertension	I10, I11, I12, I13, I15, I16
Diabetes mellitus	E10, E11
Renal diseases	N00, N01, N02, N03, N04, N05, N07, N11, N14, N17, N18, N19, Q61
Liver diseases	B15, B16, B17, B18, B19, K70, K71, K72, K73, K74, K76
Dyslipidemia	E78
Cancer	C
Pulmonary diseases	I27.8, I27.9, J40 – J47, J60 – J67, J68.4, J70.1, J70.3
**Neurological diseases**	
Dementia	F01, F02, F03, G30, G31.0
Delirium	F05
Parkinson's disease	G20
**Psychiatric disease**	
Anxiety	F40, F41
Schizophrenia	F20, F21, F22, F23, F24, F25, F28, F29
**Mood disorders**	
Unspecified non-organic psychosis	F29
Manic episode	F30
Bipolar affective disorder	F31
Depressive disorder, not elsewhere classified	F32.9
Recurrent depressive disorder	F33
Personal history of other mental and behavioral disorders	Z86.5
Organic personality disorder	F07.0
**Other psychiatric disease**	
Other persistent mental disorders due to conditions classified elsewhere	F06.0
Other specified mental disorders due to brain damage and dysfunction and to physical disease	F06.8
Unspecified mental disorder due to brain damage and dysfunction and to physical disease	F06.9
Organic delusional [schizophrenia-like] disorder	F06.2
Unspecified organic or symptomatic mental disorder	F09
